# The Utility of Invasive Hemodynamic Assessment in Diagnosing Constrictive Pericarditis: A Case Report

**DOI:** 10.7759/cureus.63454

**Published:** 2024-06-29

**Authors:** Fawaz Mohammed, Sajjad Haider, Jacqueline Dawson Dowe, Muhammad Akbar, Mohammad Abdul-Waheed

**Affiliations:** 1 Internal Medicine, University of Kentucky College of Medicine, Bowling Green, USA; 2 Cardiology, University of Kentucky College of Medicine, Bowling Green, USA; 3 Internal Medicine, Medical Center at Bowling Green, Bowling Green, USA

**Keywords:** fluoroscopy, pericardial effusion, right heart catheterization, viral pericarditis, constrictive physiology

## Abstract

Pericarditis leading to constrictive physiology is rarely diagnosed given its vague presentation. Abnormal diastolic filling from a stiff pericardium brings about signs and symptoms consistent with right-sided heart failure. We report the case of a 57-year-old female who presented with worsening shortness of breath and signs of volume overload. Chest computed tomography showed evidence of pericardial calcifications with pericardial effusion. Further evaluation with right heart catheterization suggested findings diagnostic of constrictive pericarditis.

## Introduction

The pericardium is formed by two layers comprising tissue consisting of fibroelastic tissue. Constrictive pericarditis (CP) occurs when elasticity of the pericardium is compromised leading to impaired diastolic filling [1]. Idiopathic or viral causes of CP have been reported commonly in developed countries. Less commonly described etiologies are from a prior history of heart surgery or radiation exposure [2]. Diagnosing CP is often challenging given the obscure nature of the disease. Signs and symptoms tend to overlap with valvular disorders, restrictive cardiomyopathy (RCM), or right-sided heart failure broadening the differential diagnosis. Multi-modality imaging is of utility with invasive hemodynamic monitoring being the gold standard for diagnosis [3]. Herein, we report a case of a 57-year-old female who presented to the hospital with worsening shortness of breath found to have diagnostic findings of CP with right heart catheterization (RHC).

## Case presentation

**Figure 1:**
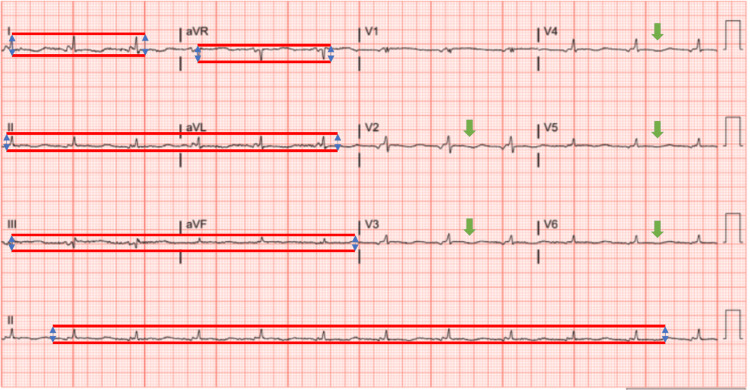
Electrocardiogram findings Electrocardiogram showing normal sinus rhythm with less than 5 mm QRS complexes vertically in limb leads (I, II, III, aVR, aVL, and aVF) meeting low voltage criteria. Also demonstrating non-specific ST-T wave changes in the anterolateral leads (green arrows).

A 57-year-old female presented to the hospital with chief complaints of shortness of breath that had worsened over a month. She had a past medical history of hypertension, dyslipidemia, obstructive sleep apnea, and asthma. She reported that her dyspnea was only on exertion initially, however, had progressed to dyspnea at rest which prompted her to come to the hospital for further evaluation. She also complained of symptoms of paroxysmal nocturnal dyspnea and orthopnea she would have every other day. She denied any associated rashes, joint swelling, joint pains, hemoptysis, hematuria, or gastrointestinal bleeding. She did report having an upper respiratory infection a couple of months back with low-grade fevers, cough, and congestion. She noted that shortly thereafter her symptoms of dyspnea began. Blood pressure on presentation was 116/72 mmHg with a heart rate of 77/min. Physical examination on admission showed 2+ bilateral pitting edema in ankles with bibasilar crackles on chest auscultation. Laboratory results showed hemoglobin levels of 11.6 g/dL (12-16g/dL), potassium of 2.8 mmol/L (3.5-5.1 mmol/L) and normal creatinine levels. N-terminal pro-B-type natriuretic peptide (NT-proBNP) was elevated at 1390 pg/L (normal value <125 pg/L). The electrocardiogram revealed nonspecific ST-T changes (Figure 1). Chest X-ray showed multichamber cardiac enlargement with central vascular congestion and bilateral small pleural effusions consistent with volume overload.

**Figure 2:**
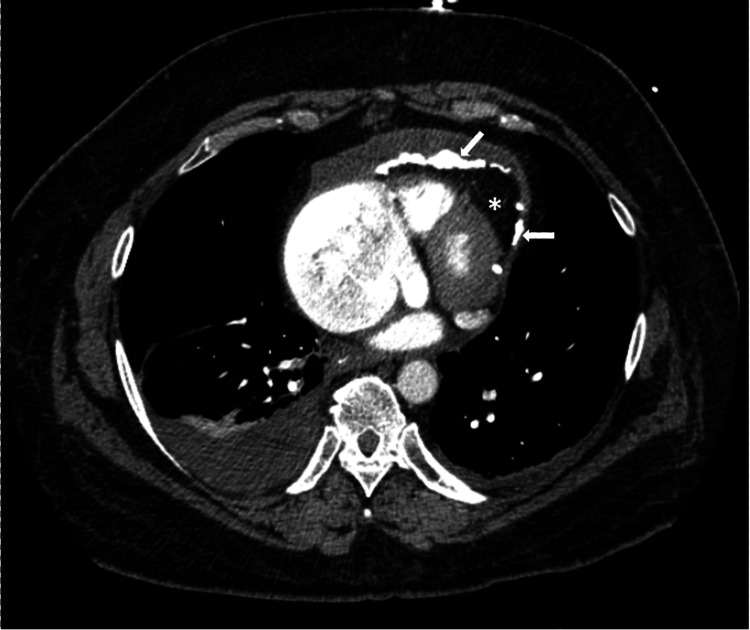
Chest computed tomography Chest computed tomography demonstrating pericardial effusion (asterisk) and pericardial calcifications (arrows).

On hospital day 2, the patient complained of chest pain. Troponin levels were within normal limits and EKG showed no acute changes. She had elevated D-dimer levels of 2.74 (0-0.49 ug/mL FEU) and a CT pulmonary angiogram (CTPA) was performed to rule out pulmonary embolism. CTPA was negative for pulmonary embolism, however, showed bilateral pleural effusions and pericardial effusion with pericardial calcifications (Figure 2).

**Figure 3:**
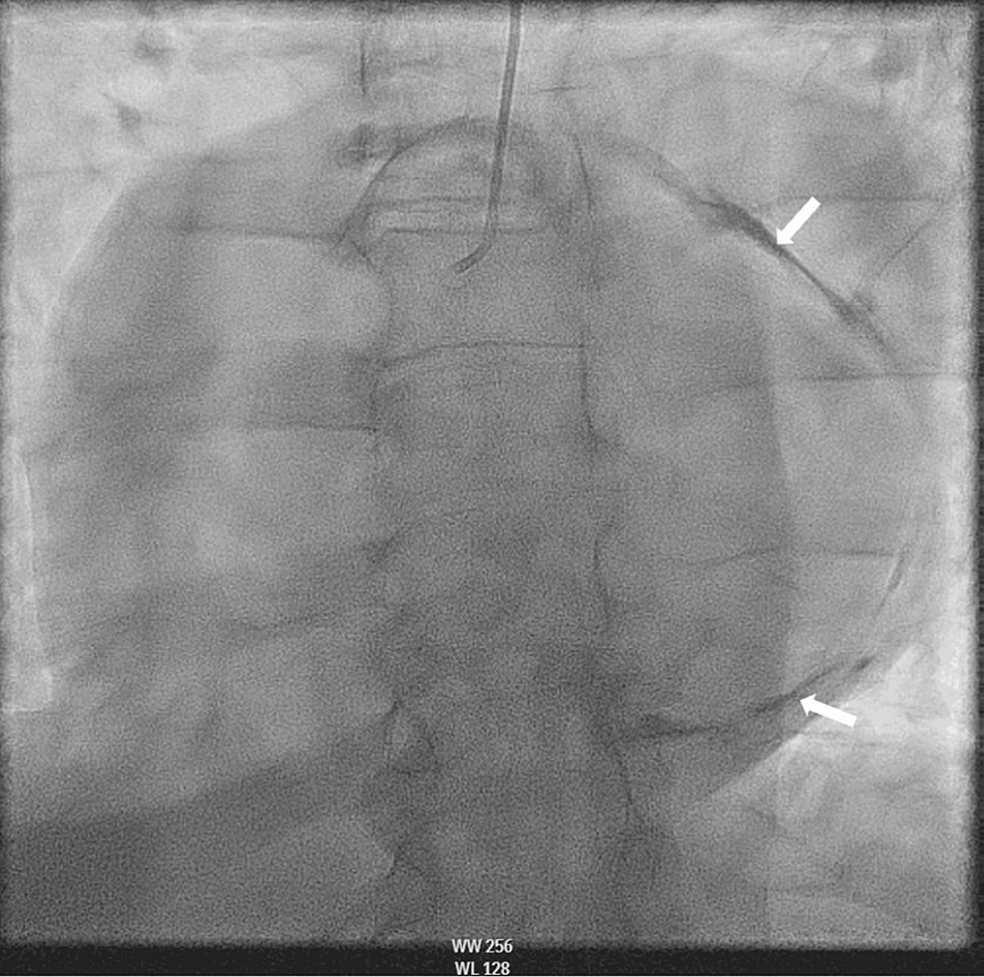
View of the pericardium with fluoroscopy Fluoroscopy showing eggshell calcifications of the pericardium (arrows).

**Figure 4:**
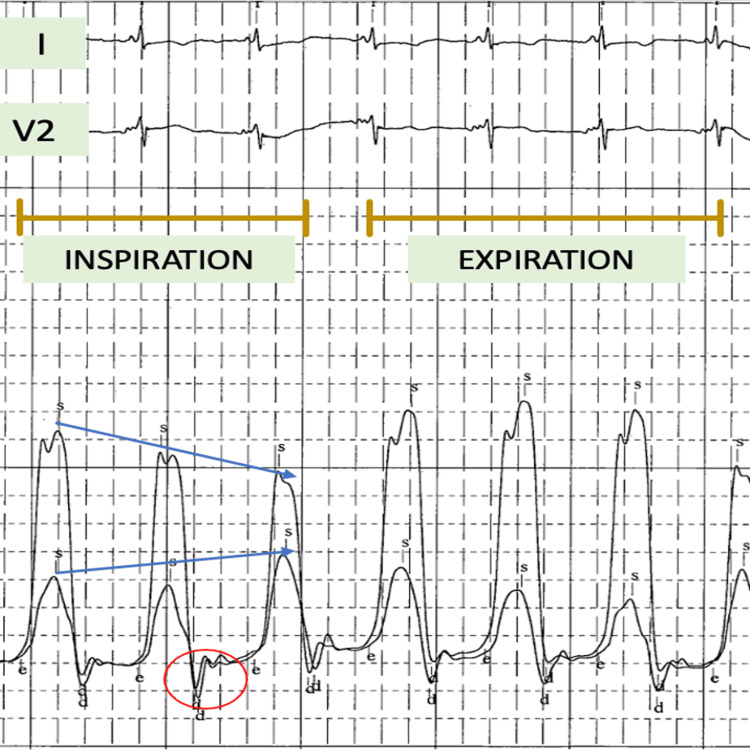
Right heart catheterization findings Right heart catheterization with simultaneous left ventricular and right ventricular pressure tracing showing discordant pressure change during inspiration (arrows), square root sign (red circle).

Fluoroscopy revealed dense calcifications (Figure 3). A RHC was then performed to measure biventricular pressures invasively simultaneously. RHC showed discordance in interventricular pressures (Figure 4). With these constellations of findings, the diagnosis of CP was made. Autoimmune work-up including erythrocyte sedimentation rate, rheumatoid factor, and anti-nuclear antibody was within normal limits. The patient was then referred to cardiothoracic surgery and has undergone surgical pericardiectomy. She tolerated the procedure well and was discharged to a skilled inpatient facility for rehabilitative purposes.

## Discussion

Our case further adds to the body of evidence of CP presenting with signs and symptoms of volume overload. Diagnosing CP remains a challenge as signs and symptoms tend to overlap with various etiologies of congestive heart failure warranting meticulous history taking and physical examination. Physical examination findings of lower extremity edema and enlargement of the liver are found in more than 50% of cases [4]. Ascites from liver disease in CP are frequently reported and are often misjudged as nonalcoholic steatohepatitis as a cause of cirrhosis [5]. Jugular venous pressure (JVP) elevation remains the most important finding as it is typically not found in chronic cases of liver dysfunction. Given its non-specific findings, multiple investigative modalities including echocardiography, cardiac magnetic resonance (CMR), and invasive hemodynamic monitoring are required [3]. Enhanced ventricular interdependence is a decisive finding that helps differentiate CP from RCM [6]. NT-proBNP can also help differentiate CP from RCM as the latter more commonly causes elevated NT-proBNP levels as opposed to the former in which stretching of the myocardium is classically not seen [7]. Pericardial thickening in CP causes typical finding of septal bounce that occurs with respiratory variation in which inspiration causes increased filling in the right ventricle, however, the high left ventricular diastolic pressure in CP leads to reduced pressure gradients that decrease left ventricular filling [3]. The opposite of this phenomenon occurs in expiration. Normally, these interventricular variations are negligible but are exaggerated in CP due to a non-compliant ventricle. Calcifications seen on plain radiography can clue physicians into CP; however, this finding is present only in a quarter of patients [8]. Multidetector cardiac computed tomography (MDCT) can help ascertain the extent of pericardial calcification and can also help recognize concomitant lung pathology in patients with prior radiation exposure or cardiac surgery although the absence of pericardial thickening does not exclude the diagnosis [9]. The “square root sign” or “dip and plateau pattern” on invasive hemodynamic monitoring seen on the right ventricle and left ventricle pressure tracings along with equalization of diastolic pressure tracings of the four chambers is also seen in CP although the specificity is relatively low [3].

CMR can help identify inflammation in the active stage where anti-inflammatories can be used as opposed to chronic CP where pericardiectomy is performed [10]. CMR sequences can detect constrictive physiology with inspiratory leftward septal excursion along with pericardial thickening having a sensitivity of 100% and specificity of 90% to diagnose CP [11]. The etiology of CP in our case has not been determined granted the patient did have symptoms of a viral prodrome which may have caused pericarditis of viral etiology, the most common cause of CP [3]. Our patient did not have a history of radiation exposure and her autoimmune workup was negative. Progression of CP depends on the etiology of CP with bacterial pericarditis having the highest rate of progression [2]. Reports have suggested that in cases where CP is reversible, it is often associated with systemic and pericardial inflammation. The use of anti-inflammatories in these cases can help reduce inflammation; leading to the resolution of signs and symptoms [12]. Medical management often comprises nonsteroidal anti-inflammatory drugs (NSAIDs) and colchicine. In patients who fail to respond to NSAIDs, corticosteroids may be used as second-line agents [3]. The use of interleukin-1 (IL-1) antagonists has also been proposed in cases of refractory recurrent pericarditis [3]. Chronic CP when left untreated has a poor prognosis with pericardiectomy having a mortality rate of approximately 2% in the hospital [13].

## Conclusions

Suspicion for CP should be maintained in patients presenting with signs of volume overload. It is often a diagnostic conundrum given overlapping signs and symptoms with other etiologies of congestive heart failure. A multimodality approach consisting of echocardiography and CMR can help recognize CP. When the diagnosis is equivocal, invasive hemodynamic assessment is indicated to confirm the diagnosis.
